# Supplementary material for: Novel Vaccine against Pathological Pyroglutamate-Modified Amyloid Beta for Prevention of Alzheimer’s Disease
Source: Int J Mol Sci. 2023 Jun 6;24(12):9797. doi: 10.3390/ijms24129797 (PMC10298272; doi:10.3390/ijms24129797)
Supplement: Supplementary file 1 [file ijms-24-09797-s001.zip › ijms-2399310-supplementary.pdf]

Supplementary Materials

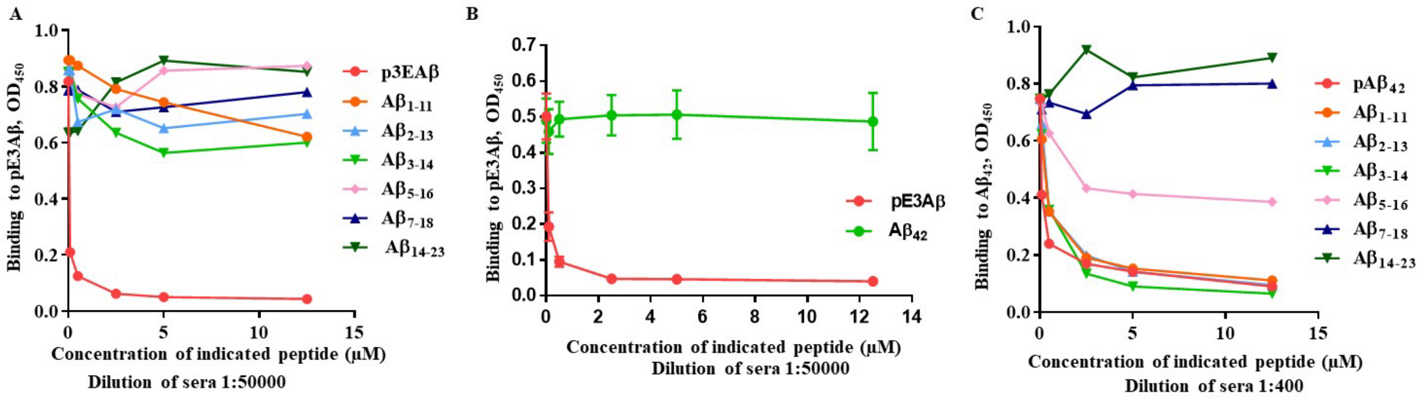

Figure S1. Binding inhibition studies with indicated concentrations of various short peptides compassing $A\beta_{42}$ using competition assay. The sera from mice immunized with pE3Aβ-based vaccine were diluted and preincubated for 1 hour at 37°C with the aforementioned peptides before binding to pE3Aβ-coated plates (**A**,**B**) and $A\beta_{42}$-coated plates **(C)** to characterize the epitope of non-specific antibodies. Note, that sera were used at a much lower dilution for the third panel.

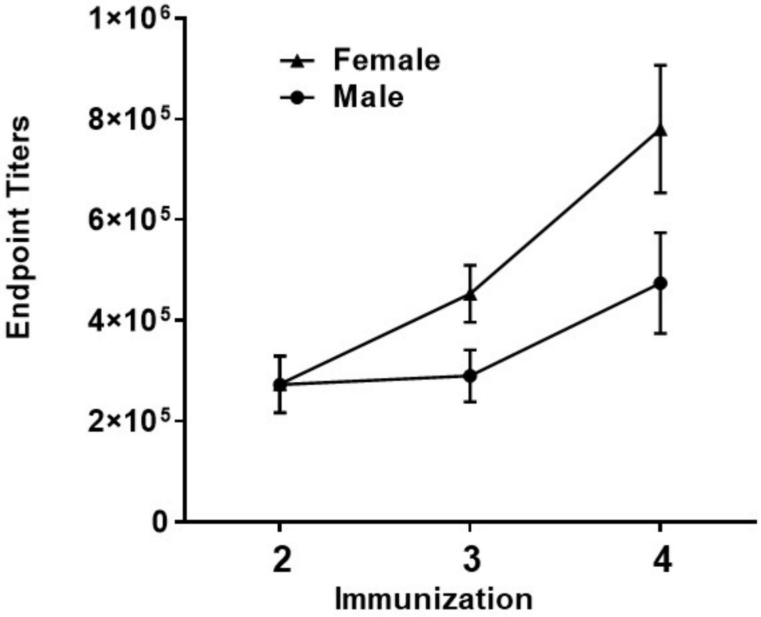

Figure S2. Dynamics of humoral immune responses in 5XFAD mice measured by ELISA against the target peptide.

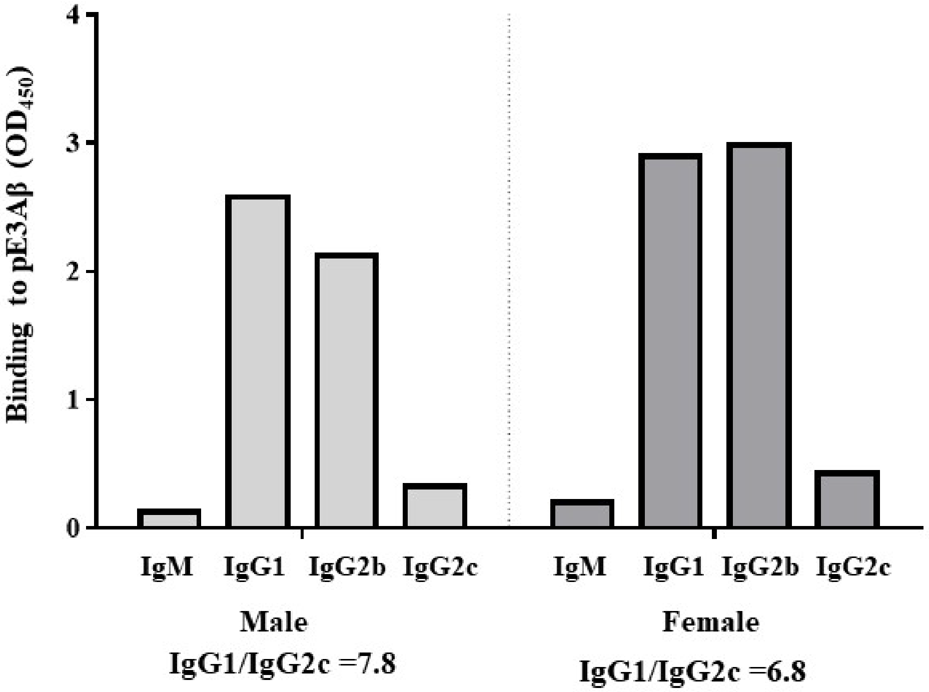

Figure S3. Immune response isotyping of the pE3Aβ-based vaccine. Isotypes of antibodies were analyzed in pooled sera collected after the 3rd immunization, at dilution 1:1000.

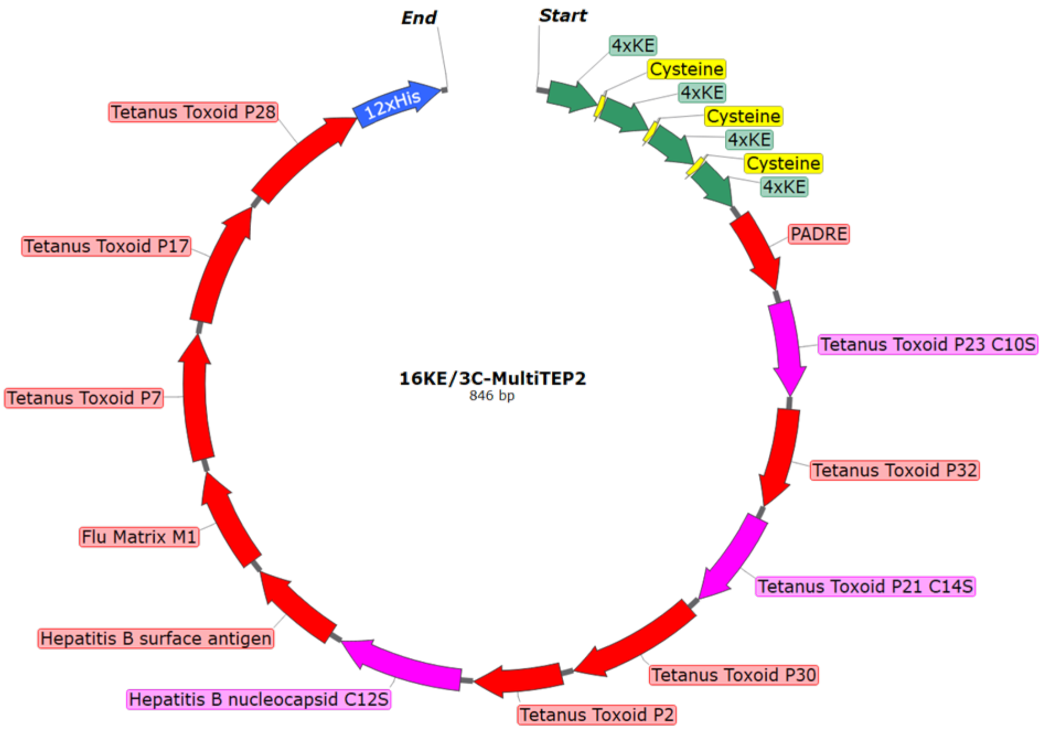

Figure S4. MultiTEP-based carrier minigene. The T helper epitopes which have been mutated to remove cysteines are labeled in magenta with the position of each mutation included in the label. The N-terminal conjugation region is composed of four zwitterionic KEKEKEKE repeats flanking three cysteines for reduced steric hindrance and increased solvent access. The C-terminus contains a 12-histidine tag for purification via IMAC.

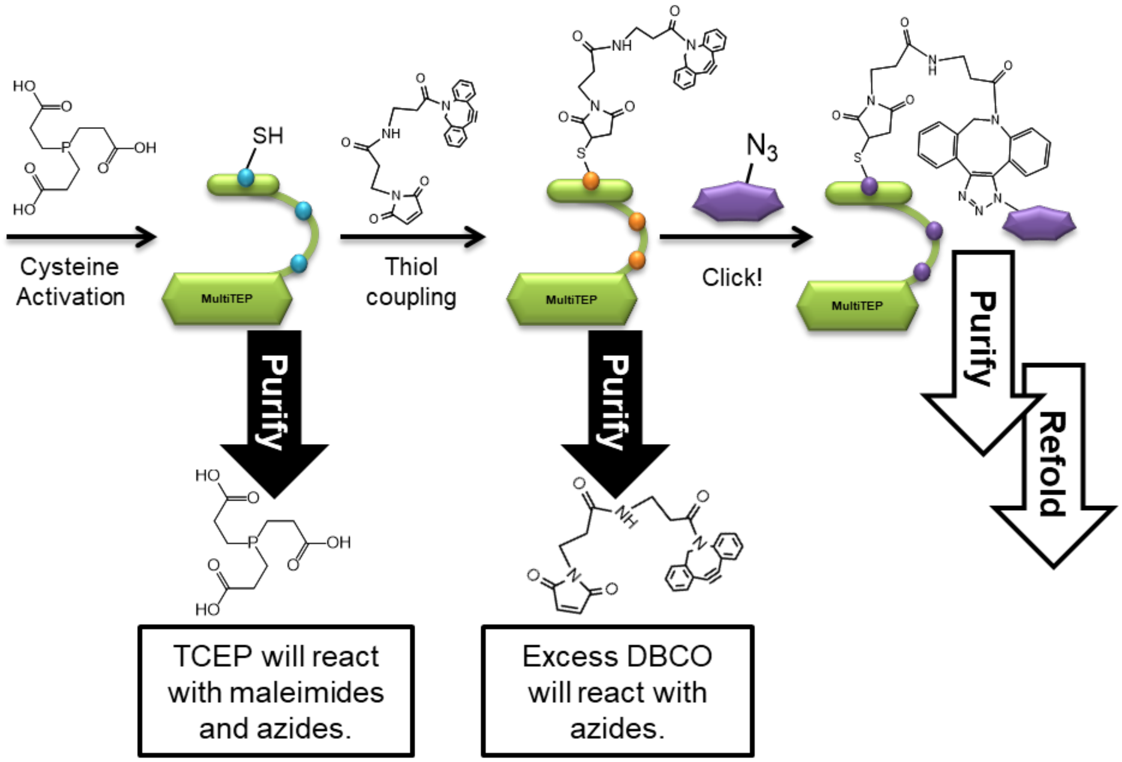

Figure S5. Preparation of the peptide-carrier conjugate. The schematic above illustrates the steps involved in the preparation of MultiTEP-based conjugate vaccines. The first step is tris carboxyethyl phosphine (TCEP) -mediated reduction of cysteines in order to obtain active thiols. The excess phosphine was removed by dialysis to avoid interference with downstream processes. The thiols were then labeled with a strained alkyne (DBCO) using thiol-ene Michael addition with a DBCO-Maleimide bifunctional crosslinker. Another dialysis was done to remove the excess cross-linker, and the protein labeled with DBCO moieties was reacted with azide-labeled pE3Aβ$_{3-11}$ via copper-free click chemistry. In order to avoid steric hindrances during the reactions, all the steps were carried out in the presence of 6 M guanidinium hydrochloride. The product was then purified from excess peptide and refolded by gradual dialysis against PBS.
